# Prediction of Clinical Outcomes in Psychotic Disorders Using Artificial Intelligence Methods: A Scoping Review

**DOI:** 10.3390/brainsci14090878

**Published:** 2024-08-29

**Authors:** Jing Ling Tay, Kyawt Kyawt Htun, Kang Sim

**Affiliations:** 1 West Region, Institute of Mental Health, Buangkok Green Medical Park, 10 Buangkok View, Singapore 539747, Singapore; 2 Institute of Mental Health, Buangkok Green Medical Park, 10 Buangkok View, Singapore 539747, Singapore; kkhtun111@gmail.com; 3 Yong Loo Lin School of Medicine, National University of Singapore, 10 Medical Drive, Singapore 117597, Singapore; 4 Lee Kong Chian School of Medicine, Nanyang Technological University, Clinical Sciences, Building, 11 Mandalay Road, Level 18, Singapore 308232, Singapore

**Keywords:** artificial intelligence, prognosis of psychotic disorders, schizophrenia, schizoaffective disorder, machine learning, psychiatric disorders

## Abstract

Background: Psychotic disorders are major psychiatric disorders that can impact multiple domains including physical, social, and psychological functioning within individuals with these conditions. Being able to better predict the outcomes of psychotic disorders will allow clinicians to identify illness subgroups and optimize treatment strategies in a timely manner. Objective: In this scoping review, we aimed to examine the accuracy of the use of artificial intelligence (AI) methods in predicting the clinical outcomes of patients with psychotic disorders as well as determine the relevant predictors of these outcomes. Methods: This review was guided by the PRISMA Guidelines for Scoping Reviews. Seven electronic databases were searched for relevant published articles in English until 1 February 2024. Results: Thirty articles were included in this review. These studies were mainly conducted in the West (63%) and Asia (37%) and published within the last 5 years (83.3%). The clinical outcomes included symptomatic improvements, illness course, and social functioning. The machine learning models utilized data from various sources including clinical, cognitive, and biological variables such as genetic, neuroimaging measures. In terms of main machine learning models used, the most common approaches were support vector machine, random forest, logistic regression, and linear regression models. No specific machine learning approach outperformed the other approaches consistently across the studies, and an overall range of predictive accuracy was observed with an AUC from 0.58 to 0.95. Specific predictors of clinical outcomes included demographic characteristics (gender, socioeconomic status, accommodation, education, and employment); social factors (activity level and interpersonal relationships); illness features (number of relapses, duration of relapses, hospitalization rates, cognitive impairments, and negative and disorganization symptoms); treatment (prescription of first-generation antipsychotics, high antipsychotic doses, clozapine, use of electroconvulsive therapy, and presence of metabolic syndrome); and structural and functional neuroimaging abnormalities, especially involving the temporal and frontal brain regions. Conclusions: The current review highlights the potential and need to further refine AI and machine learning models in parsing out the complex interplay of specific variables that contribute to the clinical outcome prediction of psychotic disorders.

## 1. Introduction

Up to 3% of the worldwide population experiences a psychotic disorder once in their lifetime [1]. Psychotic disorders form a cluster of major psychiatric conditions, including schizophrenia spectrum conditions, delusional disorder, and brief psychotic disorder [2]. These psychiatric conditions have a diverse range of symptoms such as disorganization, and positive (delusions and hallucinations) and negative symptoms (alogia, avolition, anhedonia, withdrawal, and restricted emotional expression) [3]. Positive symptoms are attributed to dopamine hyperactivity in the mesolimbic pathway, while negative symptoms are attributed to dopamine deficiency in the mesocortical pathway [4]. Dysfunction in glutamate and serotonin GABAergic signaling has also been associated with symptoms of schizophrenia [4,5]. These distressing symptoms can be associated with a significant and heterogeneous impact on the physical and psychosocial functioning within these individuals [6,7] as well as treatment responses [8,9,10]. Thus, pertinent clinical outcomes include symptom severity, illness course, and social functioning [11,12]. Prompt personalized and effective treatments can ameliorate the symptoms and improve the quality of life [13,14] of these potentially disabling mental conditions. As such, there is a need to better understand the predictors of clinical outcomes in individuals with psychotic disorders to identify illness subgroups and optimize treatment strategies early on. While clinicians could identify specific factors contributing to better prognosis of psychotic disorders, the prediction of prognosis using artificial intelligence (AI) methods is not commonly used within clinics [15]. Whilst existing reviews focused on the use of AI to detect and diagnose mental conditions [16,17,18] or recommend certain treatments [18], there is, to date, no review of extant studies pertaining to the use of AI in the clinical prognostication of psychotic disorders.

Artificial intelligence (AI) is defined as the ‘science and engineering of making intelligent machines, especially intelligent computer programs’ [19]. These programs use technologies such as machine learning, deep learning, text mining, computer vision, speech recognition, and natural language generation to gather and/or use data to predict, recommend, or decide, with varying levels of autonomy, the best action to achieve specific goals [20,21]. Within commonly employed machine learning technologies, approaches include supervised and unsupervised algorithms. Supervised learning uses and processes labelled data, whilst unsupervised learning manages unlabeled data [22,23]. Of note, AI methods using machine learning methods can process variable amounts of data and have been employed in the prediction of clinical status and outcomes of different conditions. For example, larger databases were used to predict (a) psychiatric symptoms in young adults [24] and (b) mental health crisis from electronic records [25], while smaller data databases examined specific measures such as neuroimaging scans [26,27] in predicting psychotic symptoms or wearable data in predicting aggression [28].

For psychotic disorders, recent studies have examined the use of machine learning methodologies in predicting the clinical outcomes, which included changes in symptomatology, treatment outcomes [27,29], illness trajectory [26,30], personal functioning [31,32], and quality of life [32,33]. Several studies have examined specific predictors of these clinical outcomes; for example, studies had found that factors associated with symptom remission in psychotic disorders included a short duration of untreated illness, less severe negative symptoms, better neurocognitive function, higher baseline education level, higher education, and better psychosocial functioning [31,34,35]. Conversely, factors identified as being associated with a bad prognosis of psychotic disorders included male gender, younger age of onset, lower education, long duration of untreated psychosis, negative symptoms, cognitive symptoms, manic symptoms, positive family history of psychotic disorder, poor premorbid functioning, poor help-seeking behaviors, lack of response to antipsychotics, poor diet, limited social support, and drug and cigarette use [31,36,37,38,39]. However, not all the above factors are consistently associated with specific prognoses of psychotic disorders and the interplay between these factors remains inconclusive.

Based on extant data, the aim of this scoping review was to evaluate the predictive accuracy of machine learning methods for clinical outcomes (such as symptom severity, illness course, and social functioning) and identify specific relevant predictors of such outcomes within patients with psychotic disorders.

## 2. Methodology

The methodology of this paper was guided by the Preferred Reporting Items for Systematic Reviews and Meta Analyses extension for Scoping Review [40]. The review was prospectively registered at the Open Science Framework, (osf.io/y9sz3). The research questions were as follows:

What is the predictive accuracy for clinical outcomes of patients with psychotic disorders using artificial intelligence methods?

What are the relevant predictors identified with these associated clinical outcomes in patients with psychotic disorders?

For question one, predictive accuracy was established by measures in relevant studies including area under curve (AUC), accuracy, balanced accuracy, and root mean square error (RMSE) wherever available.

### 2.1. Search Strategies

In this step, PubMed was initially searched using the keywords ‘(artificial intelligence)’ AND ‘schizophrenia’ AND ‘outcome*’ OR ‘prognosis’ to identify all relevant keywords. They were ‘(artificial intelligence)’ OR ‘(machine learning)’ OR ‘(natural language processing)’ OR ‘(neural network)’ OR ‘(data science)’ AND schizo* OR ‘(delusional disorder*)’ OR ‘(psychotic disorder*)’ AND prognosis OR outcome* OR functioning (Supplementary S1). The authors searched for relevant published studies within seven electronic databases—PubMed, Cochrane, Scopus, PsycINFO, CINAHL, EMBASE, and ScienceDirect—up to 1 February 2024. The reference lists of review papers and included articles were also reviewed for additional relevant studies.

### 2.2. Inclusion and Exclusion Criteria

Studies were included if they involved the use of artificial intelligence methods to predict clinical outcomes of psychotic disorders. Only studies published in English were included. Studies were excluded if they did not examine the stated research questions. Opinion papers, commentaries, and dissertations were excluded. Studies that were finally included in this review were reviewed by all authors [J.L.T., K.K.H., and K.S.].

### 2.3. Data Collection

In this step, key details of each included study (including authors, year of publication, study location and setting, study population, variables collected, outcomes, and main findings) were extracted and recorded in a table.

### 2.4. Quality Assessment

We additionally included a quality assessment and adopted the Joanna Briggs Institute Critical Appraisal tool for diagnostic test accuracy studies [41] within this step (Supplementary S2). Two authors [J.L.T. and K.K.H.] evaluated the methodological quality of each paper independently. Discrepancies that arose were settled through further discussions and consensus within the team.

### 2.5. Data Analysis

In addition to the table containing a summary of the salient features of the included studies, a second table detailed the predictive accuracy values according to the ML method employed and the clinical outcome examined within each study.

## 3. Results

### 3.1. General Features of Studies

**Figure 1:**
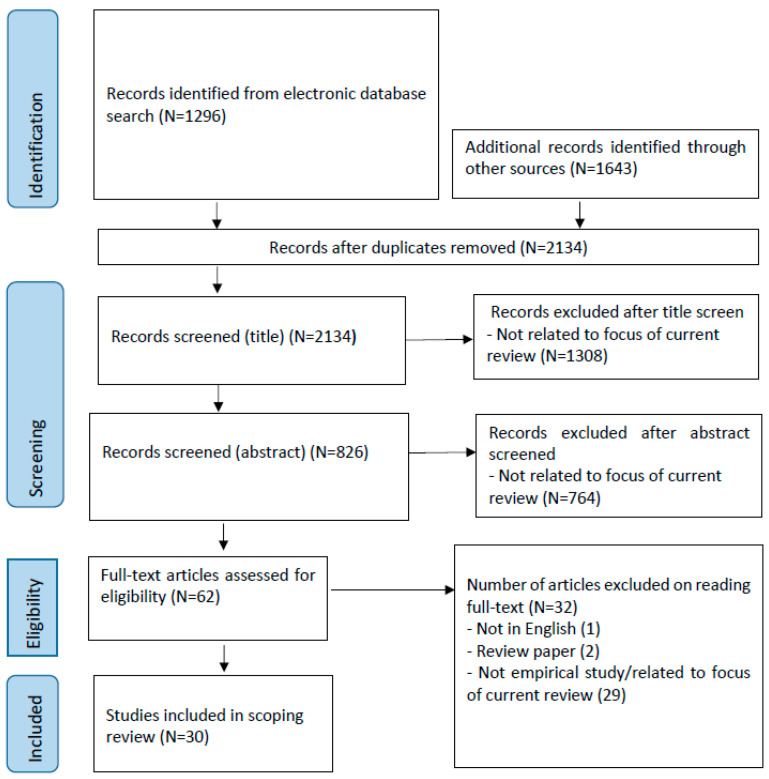
PRISMA chart.

Overall, out of 2134 studies screened, we included 30 studies in this review (see Figure 1 for a PRISMA flowchart showing the search process and final number of included papers). The rating scores of the quality assessment (see Supplementary S2) ranged from four to seven, with 19 studies scoring between four and six. Questions regarding index, reference, and interval results were deemed not applicable.

The details of the included studies are summarized in Table 1 (condensed version) and Supplementary S3 (expanded version). The included studies were conducted mainly in the last 5 years (n = 25, 83.3%) and in the West (n = 19, 63%) and Asia (n = 11, 37%), with the following breakdown: Australia (n = 1) [42], Greece (n = 1) [43], Denmark (n = 2) [29,44], France (n = 1) [45], the Netherlands (n = 1) [46], Israel (n = 1) [47], the United States (n = 4) [48,49,50,51], the United Kingdom (n = 3) [39,52,53], China (n = 7) [26,27,30,54,55,56,57], and Taiwan (n = 3) [33,58,59]. Four studies recruited participants from more than one country. They were recruited from (1) the Netherlands and Belgium [60]; (2) Denmark, England, and Scotland [32]; (3) Israel and 14 European countries [31]; and (4) 14 European countries, Israel, and Australia [61]. The data from one study [62] were obtained from the CATIE trial, while a final study [63] was obtained from an open source website.

Participants included patients with schizophrenia, schizoaffective disorder, schizophreniform disorder, first-episode psychosis, and schizotypal and/or delusional disorders. Sample sizes ranged from 11 to 32,277. The variables evaluated in the prediction model included demographic features, personal and social history (such as accommodation, education history, employment, and family history), genetic data, cognitive functioning, MRI data, medication history, and mobile sensing data.

### 3.2. Predictive Accuracy and AI Models Used for Prediction of Clinical Outcomes

In terms of predictive accuracy, most studies reported the area under the curve (AUC), accuracy, or balanced accuracy values (Table 2). AUC is a measure of accuracy on a receiver operator characteristics (ROC) curve and distinguishes between true negative and positive cases [64] for the clinical outcomes within this review. The AUC scores are interpreted as ≥0.9 (outstanding), 0.80 to <0.90 (excellent), 0.7 to <0.8 (acceptable), and 0.50 to <0.70 (poor). See Supplementary S4. Overall, a wide range of predictive accuracy was observed, with AUCs from 0.58 [61] to 0.95 [26]. The second metric accuracy evaluates how often a machine learning classification model predicts a data point accurately. It is the division of correct predictions by the total prediction number. Another metric, balanced accuracy, is an adjusted metric that is used for imbalanced datasets, and the formula is (sensitivity + specificity)/2. Accuracy scores are interpreted as follows: >90% (very good), 70–90% (good), 60–70% (acceptable), and <60% (poor) [65]. Overall, the range of accuracy/balanced accuracy varied from 48% [44] to 89% [26]. In addition, two studies [33,58] utilized the root mean square error (RMSE) as an evaluation measurement. RMSE measures the accuracy of regression machine learning models. It is the standard deviation of prediction errors, which illustrates the concentration of the data around the line of best fit [66]. The closer the RMSE value is to zero, the more accurate the model is.

**Figure 2:**
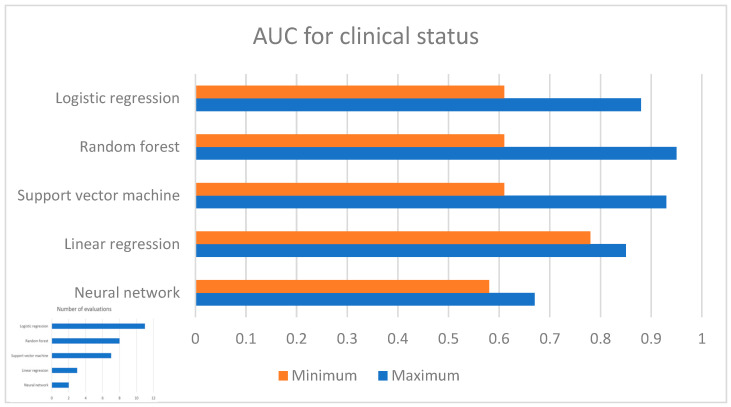
AUC for clinical status in psychotic disorders.

In terms of the main AI models used, the most common approaches were support vector machine, random forest, logistic regression, and linear regression models. Support vector machine achieved an AUC of 0.61–0.93 (poor to outstanding), accuracy values of 50–85.03% (poor to good), and an RMSE of 6.44–10.08. The random forest method achieved an AUC of 0.61–0.95 (poor to outstanding), an accuracy of 49–89% (poor to good), and an RMSE of 7.16–10.5. Logistic regression achieved an AUC 0.61–0.88 (poor to excellent) and accuracy values of 50.3–67% (poor to acceptable). Linear regression achieved an AUC range of 0.78–0.85 (acceptable to excellent), an accuracy value of 67.7–72.1% (acceptable to good), and an RMSE of 6.56–9.70. No specific approach outperformed the other approaches consistently across the studies. For the AUC values, the three highest scores were 0.95 [26], 0.93 [56], and 0.88 [52]. Regarding accuracy scores, the three highest scores were 89% [26], 85.03% [54], and 82.5% [27]. Figure 2 shows the range of AUCs for the respective algorithms.

### 3.3. Predictors of Prognosis

The main clinical outcomes of interest within the 30 studies included symptomatic change, illness course, treatment response, quality of life, and psychosocial functioning. Nine studies examined predictors of negative outcomes, including relapse of psychotic condition, non-response to treatment, treatment resistance, and poor overall functioning [31,39,43,45,46,47,51,60,62]. Nine studies examined the predictors of better prognosis, including remission (symptom, point, and period); symptom reduction; response to treatment; quality of life; and social, vocational, and overall functioning [31,32,44,47,52,55,58,59,61]. Twelve studies examined brain structure using MRI data in predicting prognosis [26,27,30,42,48,49,50,53,54,56,57,63]. One study evaluated the prediction of treatment response [29], and another study examined specific genes in predicting better quality of life and functioning [33], while a final study evaluated data from a mobile sensing system [51].

#### 3.3.1. Predictors of Negative Outcomes

Negative outcomes in this segment included relapse; symptom non-remission; or poor social, vocational, or global functioning.

##### Demographic Data

In terms of demographic data, male gender [31,61], black ethnicity [39], older [60,61] and younger ages [39], higher body mass index [61], less education [46], unemployment [61], financial difficulties [31], receiving community aid [47], accommodation problems [31,52], living alone [61], or living in a poorer neighborhood [39,46] were associated with poorer outcomes.

##### Social Factors

Social factors that were predictive of negative outcomes included poor baseline functioning [46], lack of engaging activity [31,60], poorer interpersonal relationships with accompanying psychological distress [31], childhood sexual trauma [46], and social withdrawal [32]. Likewise, total scores for the Personal Social Performance scale was identified as a predictive factor for poor outcome [43]. In addition, mobile sensing-based changes in social factors such as conversation and distance travelled detected could predict worsening of symptoms [51].

##### Illness Course and Symptoms

Recurrent relapses [31], duration of last hospitalization stay [47], presence of relapses in the past year, and high hospitalization rates were associated with poor outcomes. Similarly, substance use [32,46,61], alcohol use [52], depressive symptoms [45,60,62], aggression [45], suicidality [31,57], cognitive impairments [62], both positive [32,39,43,45,52,60,62] and negative symptoms [43,47,52,60,62], and metabolic syndrome [45] were predictive of poor outcomes. Lower baseline PANSS scores for positive symptoms, hyperactivity, conceptual disorganization [31], and lower GAF scores [43,45] were associated with poor outcomes [31].

##### Treatment

Treatments that were associated with poor outcomes included prescription of first-generation antipsychotics such as haloperidol [31,45], high antipsychotic doses [45], and low-potency antipsychotics [47].

#### 3.3.2. Predictors of Positive Outcomes

Positive outcomes in this segment included symptom remission or improved social, vocational, or global functioning.

##### Demographics

In terms of demographic characteristics, being female [55], single [55], of white ethnicity [32]; staying in a personal or family house [32,52]; and having more years of education [32,52], higher socioeconomic status [47], and current employment [31,32,55] were associated with better outcomes.

##### Social Factors

Good prognostic factors included having stable interpersonal relationships [31,32,52] and engaging in leisure activities [31].

##### Illness Course and Symptoms

Older age of onset [47], first episode, fewer relapses, shorter relapse duration, and high premorbid functioning [47,52] were associated with good outcomes [55]. Other factors associated with good outcomes included positive symptoms, depressive symptoms, PANSS excitement score [52], high baseline GAF scores [32], better cognitive functioning [44], fewer comorbidities [55], no self-harm behaviors [32], and good insight [32].

##### Treatment

Treatments including psychotherapy; use of noradrenergic antidepressants, clozapine, or electroconvulsive therapy [47]; and outpatient treatment settings [55] were associated with better prognosis in patients [47]. In a large study conducted in 16 countries that examined multiple variables, it was found that cytokines, especially cytokine IL-18, was helpful at predicting treatment response [61].

#### 3.3.3. Biological Predictors of Clinical Outcomes Based on MRI and Genotyping Data

The parahippocampal gyri, basal ganglia, cingulate and thalami were found to differentiate participants with a continuous illness course and participants with an episodic illness course [53]. Participants with a continuous illness course had volume reductions in their parahippocampal gyri [67] and larger grey matter decreases in their basal ganglia, cingulate, and thalami [53].

Low functional connectivity between the superior temporal cortex and other cortical areas were found in people experiencing psychosis [27]. Worsening positive symptoms were predicted by hypo-connectivity between the bilateral temporal poles and ventral medial prefrontal cortex, and between the right temporal parietal junction and left temporal pole. There was also hypo-connectivity between the left temporal pole, left retrosplenial cortex, left parahippocampal cortex, and other regions of the default mode network [42]. Worsening negative symptoms were predicted by hyper-connectivity between (1) the anterior medial prefrontal cortex and posterior cingulate cortex, (2) the ventral medial prefrontal cortex and left parahippocampal cortex, and (3) the left lateral temporal cortex and other regions in the left hemisphere [42]. Hypo-connectivity predicts worsening positive symptoms, while hyper-connectivity predicts worsening negative symptoms in baseline resting state connectivity within the default mode network.

At baseline, greater striatal connectivity of the posterior regions and lower striatal connectivity of the frontal regions were linked with better treatment response. Higher left anteromedial functional connectivity with the right superior frontal gyrus, right posterior insular-opercular cortex, and left precentral and postcentral gyri predicted treatment response [26]. The dynamic resting state connectivity within the default mode network had greater predictive value as compared with brain structural, clinical, or demographic features [42] in predicting treatment response. Functional connectivity between the left putamen and triangular portion of the right inferior frontal gyrus pairs also predicted treatment response [54].

Dorsolateral prefrontal cortex activation was found to be predictive of better outcomes [50]. In terms of brain structures, the brain regions that differentiated responders versus non-responders to antipsychotics included (1) grey matter volume within the left inferior frontal gyrus, (2) amplitude of low-frequency fluctuations, and (3) cortical thickness in atlas region numbers 31, 14, and 15; 119; and 157 and 42, respectively [60]. In contrast, another study found that structural MRI was not able to predict symptom remission [44]. In terms of genotyping, G72 rs2391191 and MET rs2237717 were predictive of better quality of life while rs1130233 was predictive for Global Assessment of Functioning [33].

## 4. Discussion

Our review highlights a few main findings. First, the number of studies was modest, compared to existing reviews [68,69] that evaluated prediction studies within the field of mental health. The studies were mainly published in the last five years, with relative heterogeneity of clinical outcomes examined in the different reports, including changes in severity of psychopathology, illness course, treatment response, and psychosocial functioning. Second, no specific AI approach outperformed the other approaches consistently across the studies, and an overall range of predictive accuracy was observed with an AUC from 0.58 to 0.95 and an accuracy from 48% to 89%. Third, specific predictors of the clinical outcomes included socio-demographic characteristics (gender, marital status, socioeconomic status, accommodation, education, employment, activity level, and interpersonal relationships), illness course and symptoms (number of relapses, duration of relapses, hospitalization rates, cognitive impairments, and negative and disorganization symptoms), treatment factors (prescription of first-generation antipsychotic, high doses, and clozapine; use of electroconvulsive therapy; and presence of metabolic syndrome), and structural and functional neuroimaging abnormalities involving temporal and frontal brain regions.

### 4.1. Clinical Outcomes and Predictive Variables Examined

The clinical outcomes and predictive variables in the included studies within this scoping review were diverse and comparable with extant reviews of other psychiatric disorders such as major depression and bipolar disorder [70,71,72,73]. For example, a recent review that examined AI methods in predicting outcomes of depression included only studies which examined treatment response and involved parameters including demographic, clinical, neuroimaging, genetic, and cognitive data [70]. Another review that examined individualized response to treatment modalities in depression (electroconvulsive therapy, and pharmacological and non-pharmacological treatment) utilized only neuroimaging data as predictive variables [71]. For bipolar disorder, two empirical studies had evaluated the use of demographic and clinical data to predict specifically depressive relapse [72], hospitalization, and mortality at one year [73] amongst individuals with bipolar disorder.

### 4.2. Predictive Accuracy of AI Methods for Clinical Outcomes

The common machine learning algorithms evaluated in this review were the support vector machine, random forest, logistic regression, and linear regression models. Similar to the findings from this review, the logistic regression [71], random forest [71], and especially support vector machine methods [70,71,73] were amongst the most commonly used machine learning algorithm to predict prognosis (clinical course, relapses, and treatment response) in depression and bipolar disorder, while deep learning algorithms were the least evaluated [70].

This review found that no specific AI approach outperformed the other approaches consistently. Likewise, another review of current studies using machine learning methods to predict the treatment outcomes of depression found no superiority of specific machine learning algorithm in its predictive accuracy of clinical outcome [70]. Conversely, some studies found that certain machine learning models performed better, albeit in non-psychotic disorders such as affective disorders. For example, a US study [72] examining predictors of depressive relapse in bipolar disorder found that the random forest model performed the best compared with the support vector machine, naïve bayes, multilayer perceptron, and logistic regression algorithms, whilst another US study [73] found that deep neural network performed better than a traditional machine learning algorithm (support vector machine) for outcome prediction. Conversely, the study [60] that utilized a deep neural network in this review did not perform the best. Its poor performance could be due to the excessive number of variables included within the model.

The studies within this review reported an AUC ranging from 0.58 to 0.95 and an accuracy ranging from 48% to 89%. One explanation for the wide range of reported predictive accuracy and accuracy values even within the same algorithms could be the heterogeneous nature of the predictive variables and outcome measures examined across the different studies. A recent review that explored the treatment outcomes in depression reported an average accuracy of 63% amongst eight studies of adequate quality, as compared with an average of 75% in the remaining 46 studies [70]. This same review [70] also found greater predictive accuracy for treatment resistance (accuracy 69%), as compared with response (56%) or remission (60%). Additionally, studies that incorporated MRI findings into their algorithm achieved greater predictive accuracy. This was affirmed by another review [71], which examined neuroimaging data in predicting the outcomes of depression and found a pooled AUC of 0.84, with the predictive accuracy highest for electroconvulsive therapy versus pharmacological and non-pharmacological interventions. Besides MRI findings, other objective findings such as EEG and cognitive data [29,44,74,75] achieved better predictive accuracy as compared with clinical and social data [76]. This could be because the measurements for MRI and EEG are more objective, while clinical measures, especially if self-reported such as experienced symptomatology over time, may fluctuate and be subjected to recall bias. Although clinical and social data have inherent biases, they can be strengthened with triangulation of data from reliable informants and, if complemented by other cognitive and biological factors, can potentially add to the battery of predictors of positive and negative outcomes in patients with psychotic conditions.

### 4.3. Specific Predictors of the Clinical Outcomes

Regarding socio-demographic factors, we found that female gender was associated with positive outcome [31,55]. This was congruent with the literature, which reported that male gender [77,78,79] was linked with poorer outcomes. Gender also influences the clinical presentation of psychotic disorders. Males are more likely to have more severe and negative symptoms [80]. In contrast, females tended to have later age of onset and better and more speedy responses to treatment, but the benefits decline over time after onset of illness [79]. Of note, predictors of clinical outcomes which were reported previously included pre-morbid status [81,82], marital status [83], accommodation [83], socioeconomic status [84,85], education level [36,86,87,88], employment status [82], activity engagement [89], and social support [89,90]. Although one study found that singlehood was associated with positive clinical outcome, other studies had found the opposite [83,91,92]. There are likely complex inter-relationships between these underlying predictors in influencing clinical outcomes. For example, being married provides opportunities for additional caregivers and family support [92], and social support is vital in reducing the chances of relapses in psychotic disorders [90]. Poor pre-morbid education level had been associated with a longer duration of untreated psychosis [90] and cognitive decline in schizophrenia [88], and worse cognitive functioning had also been associated with lower socioeconomic status [84] and poorer response to interventions [93]. Engaging in activities is associated with positive outcomes, likely due to its positive effects on memory, cognition, attention, depressive symptoms, and global psychosocial functioning [89,94].

In terms of clinical factors, we found that higher number and duration of relapses, hospitalization rates, cognitive deficits, and psychotic psychopathology were predictors of poor outcomes such as poor functioning, which were observed in earlier studies such as numbers of relapses, hospitalization [95,96,97], duration of relapses [90], cognitive impairments [98,99], and presence of negative [100,101,102] and disorganization symptoms [103]. In this review, we found that both depressive and positive symptoms were associated with positive and negative clinical outcomes, respectively. Earlier reports had observed that depressive symptoms are associated with poorer prognosis in schizophrenia [104,105]. One explanation could be that avolition in depression may result in poor interpersonal relationships and poor adherence to psychiatric appointments, which contribute to a poorer illness course [104]. Although positive symptoms were predictive of further hospitalizations [102], these symptoms were more responsive to pharmacotherapy compared with negative symptoms [105]. Limited evidence also found that patients with metabolic syndrome had a three times higher risk of a psychotic relapse versus patients without metabolic syndrome [106], and this could be related to inflammatory factors [107]. Future precision medicine studies could evaluate whether better treatment of metabolic syndromes translate to improved prognosis for individuals with psychotic disorders [106].

Regarding treatment factors, we found that the use of first-generation antipsychotics was predictive of poorer outcome, which was in agreement with the previous literature, which reported that the use of first-generation antipsychotics is associated with poorer cognitive functioning [108]. Although second-generation antipsychotics were not without adverse effects compared with first-generation agents [109,110], patients were more likely to be adherent to second-generation antipsychotics, which likely conferred better clinical outcomes [31]. In addition, the use of antipsychotic drugs at high doses was indicative of poorer outcomes and was also reflective of underlying antipsychotic polypharmacy [108,111,112].

We found that clozapine use and electroconvulsive therapy were associated with better outcomes. Clozapine has known efficacy in treatment-resistant schizophrenia, amelioration of psychotic psychopathology, and achievement of greater response rates as compared with other antipsychotic medications [113], which can significantly improve functioning and quality of life [114]. Likewise, electroconvulsive therapy has demonstrated effectiveness in symptom reduction, and functioning and quality of life improvements amongst people with schizophrenia and treatment-resistant schizophrenia [115]. In addition, it was noted that treatments involving psychotherapy and noradrenergic antidepressants [47] were associated with better outcomes, which may be related to the severity of the clinical status, leading to such specific management options.

Regarding neuroimaging measures as predictors, we found that structural and functional brain imaging predictors, especially involving the temporal and frontal regions, influenced the clinical outcomes. Earlier studies had reported that poorer clinical outcomes in psychotic disorders were associated with smaller cortical gray matter volumes [116]; reduced white matter integrity at prefrontal and temporal regions [117]; and reduced volumes at the prefrontal [118], lingual gryus, insula, and cerebellum regions [119,120]. Better clinical outcomes were associated with greater grey matter density at the limbic and frontal regions [121] and volume increase in the hippocampal regions [122]. Previous reports had highlighted the potential of the default node network and striatal functional integration and segregation metrics as measures of treatment response [123,124]. Consistent with the reported findings, earlier studies had noted that treatment response was predicted by poor striatal connectivity with parietal lobe structures [125] and greater functional connectivity of the posterior regions [123,126,127] within the default mode network [124,128] and the inter-hemispheric regions [129]; between the hippocampus and entire brain [130]; and between the striatal region and anterior cingulate, dorsolateral prefrontal cortex, and limbic regions [124,125].

### 4.4. Limitations and Future Research

This systematic review has some limitations. First, there was a modest number of studies with variable sizes of their samples and datasets examining the use of AI methods in predicting the clinical outcomes of psychotic disorders. Second, the included studies were heterogeneous in the clinical outcomes investigated and AI methods used, thereby limiting specific quantitative analyses. Third, the heterogeneity in the metrics used to measure the predictive accuracy of clinical outcomes constrained result interpretation.

In terms of future research recommendations, first, larger, collaborative studies are warranted to expand the anonymized datasets available for investigation for greater generalizability of the findings. Second, the continual refinements of AI methods and combinatorial adoption of methods can be utilized to examine amassed data to identify relevant predictors of clinical outcomes. Third, the inclusion of multimodal, longitudinal data including biological information such as genetic, physiological, and imaging data may shed light on the factors and interactions between the factors in influencing prognosis in psychotic disorders.

## 5. Conclusions

In conclusion, this review found that within mostly recent studies, the use of AI and machine learning methods have the potential to predict the clinical outcomes of psychotic conditions and examine specific predictors of such outcomes. Predictive accuracy was observed with AUCs from 0.58 to 0.95 (poor to outstanding). Notwithstanding the heterogeneity of clinical outcomes investigated and wide overall range of reported predictive accuracy, further innovations can refine future AI and machine learning approaches and utilize cross-sectional and longitudinal multimodal information (including clinical, cognitive, and biological data points) to disentangle the complex interplay between predictors which influence the clinical outcomes of psychotic conditions.

## Figures and Tables

**Table 1:** Details of included studies.

Authors/ Year	Clinical Outcomes	AUC If Available	Predictors of Clinical Outcomes
Ambrosen et al., 2020 [29]	Symptomatic improvement		*Best algorithms for treatment response:* *logistic regression (long term), SVM with L1 regularization (short term).*
Blessing et al., 2019 [26]	Symptomatic improvement	0.95 (random forest)	Anteromedial hippocampal functional connectivity with right superior frontal gyrus, right posterior insular–opercular cortex and left pre- and postcentral gyrus predicted treatment response.
Cao et al., 2018 [27]	Symptomatic improvement		Functional connectivity between superior temporal cortex and other cortical areas.
Cui et al., 2021 [54]	Symptomatic improvement		Twelve features (three cortical features and nine functional connections) remained in the prediction model.
Cui et al., 2021 [30]	Symptomatic improvement	0.72 (SVM)	Four features from 4019 radiomics features were identified.
Ebdrup et al., 2019 [44]	Symptomatic improvement		No variable predicted symptom remission after six weeks.
Fond et al., 2019 [45]	Psychotic relapse		High hospitalization rate, use of first-generation and higher-dose antipsychotics, metabolic syndrome, CDSS, GAF, Buss and Perry anger score, PANSS for positive and depressed subscales.
Homan et al., 2019 [48]	Symptom status		Implicated notes were in the (1) prefrontal cortices; (2) posterior cingulate cortex; and (3) the precentral, superior temporal, and middle cingulate cortex.
Kottaram et al., 2019 [42]	Psychotic symptoms at 1 year	0.78–0.85 (linear R)	Predictive factors for worsening positive symptoms included hyper-dynamism and hypo-connectivity, while predictive factors for worsening negative symptoms included hypo-dynamism and hyper-connectivity.
Koutsouleris et al., 2016 [31]	Good versus poor outcome based on GAF		Poor outcome predictors included male gender; unemployment; poor educational status; recurrent relapses; suicidality; unmet needs in CAN including relationships, activities, psychological distress, money, information, accommodation, and sexual expression; Haloperidol treatment; lower baseline scores for PANSS item positive symptoms; conceptual disorganization; and hyperactivity. Good outcome predictors include greater GAF scores, positive MANSA scores for job, leisure, friendship, and health.
Lamichhane et al., 2023 [51]	Relapse		Changes in conversation, volume, and distance travelled were predictors of relapse.
Leighton et al., 2019 [52]	Employment/education status, point, period symptom remission	0.88 (logistic R) 0.63–0.65 (logistic R)	Positive predictors were baseline functioning, white ethnicity, living with family, employment, having relationships, PANSS scores for excitement, depression, and poor rapport. Negative predictors included rented accommodation, PANSS for suspiciousness, hostility, delusions, social withdrawal, somatic concern, abstract thinking difficulty, and unusual thought content.
Leighton et al., 2019 [32]	Symptom status, vocational recovery, QOL	0.70–0.74 (logistic R)	Predictors were higher education, staying in own or parents’ home, employment, no self-harm, good social support, and insight. Negative predictors were hallucinations, unusual thought content, adolescent social withdrawal, and substance use.
Li et al., 2021 [55]	Social functioning	0.81 (random forest)	Positive predictors at 3 months included female gender; younger age; being unmarried; being employed; first episode; outpatient treatment; shorter relapse duration; lesser relapse; lower baseline social functioning score; fewer comorbidities; and more severe PANSS, CDSS, and CGI scores.
Lin et al., 2021 [58]	Social functioning, QOL		Quality of life was best predicted with the Scale for the Assessments of Negative Symptoms and 17-item Hamilton Depression Rating Scale. GAF was best predicted with a PANSS-positive item and the Scale for the Assessments of Negative Symptoms.
Lin et al., 2021 [33]	Social functioning, QOL		*M5 prime algorithm identified G72 rs2391191 and MET rs2237717 as quality-of-life predictors, while AKT1 rs1130233 predicted GAF.*
Liu et al., 2022 [56]	Symptomatic improvement	0.93 (SVM)	Reduced degree centrality was found in subcortical gray matter structures. Post treatment, changes in degree centrality correlated with PANSS changes, with negative correlations in the right and left putamens.
Magrangeas et al., 2022 [39]	Negative outcomes		Predictors of negative outcomes within 2 years included younger age, black ethnicity, staying in poorer neighborhoods, and psychotic symptoms.
Modai et al., 1995 [47]	Social functioning		Predictors of positive outcomes at 8 weeks included higher socioeconomic class; positive symptoms; and receiving psychotherapy, electroconvulsive therapy, Clozapine, or noradrenergic antidepressants. Other factors were older age onset, high premorbid level, axis II diagnosis, and frequent hospitalization. Predictors of negative outcomes at 8 weeks included negative symptoms, duration of last hospitalization stay, low potency antipsychotics, requiring community aid, resistant depression, and OCD.
Mourao-Miranda et al., 2012 [53]	Course of illness		Anatomical regions that discriminated continuous course vs. episodic course and the control included the parahippocampal gyri, basal ganglia, cingulate, and thalami.
Nijs et al., 2021 [60] services	Symptomatic improvement, social functioning		Predictors of outcomes included older age, self-harm, lack of activity, emotional withdrawal, delusions, unusual thought content, PANSS for depression, flat affect, motor retardation, lack of spontaneity, hallucinatory behaviors, suspiciousness, abstract thinking difficulty, and poor judgement and insight.
Podichetty et al., 2021 [62]	Symptomatic improvement	0.65 (random forest)	Predictors were poor attention, depression, preoccupation, volition impairment, abstract thinking difficulty, stereotyped thinking, anxiety, abnormal thought content, excitement, and observed depression.
Sarpal et al., 2016 [49]	Clinical Global Impression, symptomatic improvement	0.78 (COX R)	A total of 91 connections were associated with treatment response. Greater connectivity with striatal subdivision at posterior regions and lower striatal connectivity at frontal regions were associated with better response.
Schie, 2022 [61]	Symptomatic improvement	0.58–0.67 (neural network)	Non-remitted patients were more likely to be older, males, living alone, or unemployed, and to have higher weight and greater substance use. Amongst cytokines, cytokine IL-18 was a predictor.
Soldatos et al., 2022 [43]	Symptomatic improvement	0.68 (SVM)	Predictive factors of non-remission at 4–6 weeks included PSP and GAF scores; PANSS scores for delusions; social avoidance; passive/apathetic social withdrawal; blunted affect; emotional withdrawal; poor rapport; delusions; lack of spontaneity; and poor flow of conversation, judgement, and insight.
Smucny et al., 2020 [50]	Symptomatic improvement		Activation of the dorsolateral prefrontal cortex was the most predictive factor.
Talpalaru et al., 2019 [63]	Symptom status: (1) high; (2) positive; (3) mild	0.61–0.81 (random forest)	Paracingulate gyri and the left anterior cingulate differentiated between groups: right insula, middle temporal gyri, and left temporal poles of the superior temporal affected in groups 1 and 2; left insula affected in groups 2 and 3.
Van Hooijdonk et al., 2023 [46]	Treatment resistance	0.69 (random forest)	Predictors of poor treatment response in schizophrenia included poor premorbid functioning, not being married, younger age of illness onset, childhood sexual trauma, lower education level, greater use of substances, and staying in non-urban environments.
Wang et al., 2022 [57]	Responders versus non-responders	0.86 (gradient boosting)	Predictors associated were grey matter volume, cortical thickness, aberrant amplitude low-frequency fluctuation, cortical thickness and volume, surface area, curvature, and sulcal depth.
Wu et al., 2020 [59]	Symptomatic improvements		Predictors included age; number of hospitalizations and emergency room/clinics visits; and the use of benzodiazepines, mood stabilizers, and antiepileptics.

*Italics* = findings related to algorithms; AUC = area under the curve; CDSS = Calgary Depression Scale for Schizophrenia; CGI = Clinical Global Impression; Fup = follow-up; GAF = Global Assessment of Functioning; MANSA = Manchester Short Assessment of Quality of Life Scale; MINI = Mini International Neuropsychiatric Interview; ML = machine learning; NPV = negative predictive value; PANSS = Positive and Negative Syndrome Scale; PPV = positive predictive value; PSP = Personal Social Performance; R = regression; SWN = Subjective Well-Being Under Neuroleptic Treatment Scale.

**Table 2:** Predictive accuracy of different machine learning models.

Authors		Machine Learning Models										
	Logistic r	Support Vector Machine	Linear r	Random Forest	KNN	Bagging Ensemble	Decision Tree	NN	Naïve Bayes	AB	Gradient Boosting	COX r
Ambrosen et al., 2020 [29]												
*Diagnostic classification (Balanced accuracy)*	***63.8* *****%*******	*50.4* ***%***					***64.2* ***%*****					
*Long-term response (Balanced accuracy)*	***50.3* ***%*****	*50* ***%***		*49.7* ***%***								
Blessing et al., 2019 [26] FC (AUC)				0.95								
FC (Accuracy)				89***%***								
Cao et al., 2018 [27] CFC (Balanced accuracy)		*82.5* ***%***										
MRI (Balanced accuracy)		*57.4* ***%***										
Cui et al., 2021 [54] Functional MRI		*80.38* ***%***										
Structural MRI		*69.68* ***%***										
Functional and Structural MRI) (accuracy)		*85.03* ***%***										
Cui et al., 2021 [30]												
Dataset 1 (accuracy)		*68.36* ***%***										
Dataset 2 (accuracy)		*65.21* ***%***										
AUC		0.72										
Ebdrup et al., 2019 [44]												
Cognition (accuracy)	*62* ***%***	***64*** ***%***		*56* ***%***			*48* ***%***		*59* ***%***			
EEG (accuracy)	***66*** ***%***	*64* ***%***		*49* ***%***			*50* ***%***		*48* ***%***			
MRI (accuracy)	***67*** ***%***	*64* ***%***		***67*** ***%***			*63* ***%***		*61* ***%***			
Diffusion tensor imaging (accuracy)	***66*** ***%***	*63* ***%***		*65* ***%***			*52* ***%***		*55* ***%***			
Clinical modality (accuracy)	*62* ***%***	*60* ***%***		*64* ***%***			*56* ***%***		***67*** ***%***			
Fond et al., 2019 [45]												
Relapse (Accuracy)				*63.8* ***%***								
F/up withdrawal (Accuracy)				*52.4* ***%***								
Kottaram et al., 2019 [42]												
Positive symptoms (AUC)			0.85									
Negative symptoms (AUC)			0.83									
BPRS (AUC)			0.78									
Koutsouleris et al., 2016 [31]												
*GAF at 4 weeks (Balanced accuracy)*			*69.6*–*72.1***%**									
*GAF at 52 weeks (Balanced accuracy)*			*67.7–71.5* **%**									
Leighton et al., 2019 [52]												
*Functional status* (AUC)	0.88											
*Point remission* (AUC)	0.65											
*Period remission* (AUC)	0.63											
Leighton et al., 2019 [32]												
*Symptom recovery* (AUC)	0.70											
*Social recovery* (AUC)	0.73											
*Vocational recovery* (AUC)	0.74											
*Quality of life* (AUC)	0.70											
Li et al., 2021 [55] (AUC)				0.81								
Lin et al., 2021 [58]												
*QOLS* *(RMSE)*		6.44	6.56	7.16		**6.43**–6.44		6.49				
*GAF (RMSE)*		7.91	7.96	8.45		**7.78**–7.81		7.84				
Lin et al., 2021 [33]												
*QOLS* *(RMSE)*		8.88	8.78	9.43		**8.68**–8.71		8.87				
*GAF (RMSE)*		10.08	**9.70**	10.50		**9.70**–9.78		10.06				
Liu et al., 2022 [56] (AUC)		0.93										
Mourao-Miranda et al., 2012 [53] *(Accuracy)*		*67–70* ***%***										
Nijs et al., 2021 [60]												
*GAF (3 year) (Balanced accuracy)*		*53–69.7* ***%***										
*GAF (6 year) (Balanced accuracy)*		*54.4–69.3* ***%***										
Podichetty et al., 2021 [62](AUC)				0.65								
Sarpal et al., 2016 [49] (AUC)												0.78
Schie, 2022 [61]												
*Symptoms (4 weeks)* (AUC)								0.58				
*Symptoms (10 weeks)* (AUC)								0.67				
Soldatos et al., 2022 [43](AUC)		0.68										
Smucny et al., 2020 [50] *(Accuracy)*	***63.7*** ***%***	*63.6* ***%***		*63.4* ***%***	*60.8* ***%***		*66.7* ***%***	***70* ***%*****	*67.4* ***%***	*62.9* ***%***		
Talpalaru et al., 2019 [63]												
*Schizophrenia* vs. *c* (AUC)	0.69	0.71		**0.75**								
*High symptoms* vs. *c* (AUC)	0.74	0.80		**0.81**								
*Positive symptoms* vs. *c* (AUC)	0.61	0.61		0.61								
*Mild symptoms* vs. *c* (AUC)	0.65	**0.78**		0.63								
Van Hooijdonk et al., 2023 [46] (AUC)				0.69								
Wang et al., 2022 [57] Structural MRI (AUC)											0.86	

*Italic* = balanced accuracy/accuracy; bold = best-performing algorithm. Abbreviations: AB = AdaBoost; BPRS = Brief Psychotic Rating Scale; c = control; CFC = correlational functional connections; FC = functional connectivity; f/up = follow-up; GAF = Global Assessment of functioning Scale; J48 = J48 decision tree; KNN = K nearest neighbor; NN = neural networks; MI= mutual information of the blood oxygen level-dependent signals; QOLS = Quality of Life Scale; RMSE = root mean squared error; r = regression; vs. = versus.

## Data Availability

No new data were created or analyzed in this study.

## Supplementary Materials

The following supporting information can be downloaded at https://www.mdpi.com/article/10.3390/brainsci14090878/s1, Supplementary S1: Search keywords; Supplementary S2: Quality assessment; Supplementary S3: Details of included studies (expanded version); Supplementary S4: Predictive accuracy interpretation.
