# Supplementary material for: Prediction of Clinical Outcomes in Psychotic Disorders Using Artificial Intelligence Methods: A Scoping Review
Source: Brain Sci. 2024 Aug 29;14(9):878. doi: 10.3390/brainsci14090878 (PMC11430394; doi:10.3390/brainsci14090878)
Supplement: Supplementary file 1 [file brainsci-14-00878-s001.zip › brainsci-3167918-supplementary.pdf]

Supplementary S1. Search keywords

Databases	Keywords
**CINAHL**	AB ((Artificial intelligence) OR (machine learning) OR (natural language processing) OR (neural network) OR (data science)) AND AB (schizo* OR (delusional disorder*) OR (psychotic disorder*)) AND AB (prognosis OR outcome* OR functioning)
**Cochrane Library**	‘(artificial intelligence)’ OR ‘(machine learning)’ OR ‘(natural language processing)’ OR ‘(neural network)’ OR ‘(data science)’ in Title Abstract Keyword AND schizo* OR ‘(delusional disorder*)’ OR ‘(psychotic disorder*)’ in Title Abstract Keyword AND prognosis OR outcome* OR functioning in Title Abstract Keyword - (Word variations have been searched)
**Embase**	‘(artificial intelligence)’ OR ‘(machine learning)’ OR ‘(natural language processing)’ OR ‘(neural network)’ OR ‘(data science)’ AND schizo* OR ‘(delusional disorder*)’ OR ‘(psychotic disorder*)’ AND prognosis OR outcome* OR functioning
**PsycInfo**	((artificial intelligence or machine learning or natural language processing or neural network or data science) and (schizo* or delusional disorder* or psychotic disorder*) and (prognosis or outcome* or functioning)).ab.
**PubMed**	(((artificial intelligence[Title/Abstract]) OR (machine learning[Title/Abstract]) OR (natural language processing[Title/Abstract]) OR (neural network[Title/Abstract]) OR (data science[Title/Abstract])) AND (schizo*[Title/Abstract] OR (delusional disorder*[Title/Abstract]) OR (psychotic disorder*[Title/Abstract]))) AND (prognosis[Title/Abstract] OR outcome*[Title/Abstract] OR functioning[Title/Abstract])
**Science Direct**	(Artificial intelligence) AND (schizophrenia OR schizoaffective disorder or delusional disorder OR psychotic disorder) AND (prognosis OR outcome OR functioning)

	(machine learning) AND (schizophrenia OR schizoaffective disorders or delusional disorders OR psychotic disorders) AND (prognosis OR outcome OR functioning)
	(natural language processing) AND (schizophrenia OR schizoaffective disorder or delusional disorder OR psychotic disorder) AND (prognosis OR outcome OR functioning)
	(neural network) AND (schizophrenia OR schizoaffective disorders or delusional disorders OR psychotic disorders) AND (prognosis OR outcome OR functioning)
	(data science) AND (schizophrenia OR schizoaffective disorder or delusional disorder OR psychotic disorder) AND (prognosis OR outcome OR functioning)
**Scopus**	(ABS ((artificial AND intelligence) OR (machine AND learning) OR (neural AND network) OR (data AND science)) AND TITLE (schizo* OR (delusional AND disorder*) OR (psychotic AND disorder*)) AND TITLE-ABS-KEY (prognosis OR outcome* OR functioning))

Supplementary S2. Quality assessment

Bias / Authors	1. Consecutive or random sampling	2. Case control design avoided	3. Prevent inappropriate exclusions	4. Interpretation of index results without knowledge of the standard	5. Pre-specificied threshold used	6. Appropriateness of reference standard in classifying identified	7. Interpretation of reference results without knowledge of standard	8. Ideal interval between index and reference results?	9. Same reference results for all patients?	10. Inclusion of all paitents in analysis	Total score
Ambrosen et al., 2020	++	++	++	NA	++	+	NA	NA	++	++	6
Blessing et al., 2019	++	+	++	NA	++	++	NA	NA	++	++	6
Cao et al., 2018	+	-	++	NA	++	++	NA	NA	++	++	5
Cui et al., 2021	+	++	++	NA	++	++	NA	NA	++	++	6
Cui et al., 2021	+	++	+	NA	++	++	NA	NA	++	++	5

Bias / Authors	1. Consecutive or random sampling	2. Case control design avoided	3. Prevent inappropriate exclusions	4. Interpretation of index results without knowledge of the standard	5. Pre-specificied threshold used	6. Appropriateness of reference standard in classifying identified	7. Interpretation of reference results without knowledge of standard	8. Ideal interval between index and reference results?	9. Same reference results for all patients?	10. Inclusion of all paitents in analysis	Total score
Edbrup et al., 2019	+	++	++	NA	++	+	NA	NA	++	++	5
Fond et al., 2019	++	++	+	NA	++	++	NA	NA	++	+	5
Homan et al., 2019	+	++	++	NA	++	++	NA	NA	++	++	6
Kottaram et al., 2019	+	++	++	NA	++	++	NA	NA	++	++	6
Koutsouleris et al., 2016	+	++	+	NA	++	++	NA	NA	++	+	4
Lamichhane et al., 2023	+	++	+	NA	++	++	NA	NA	++	+	4
Leighton et al., 2019	+	++	+	NA	++	++	NA	NA	++	++	5

Bias / Authors	1. Consecutive or random sampling	2. Case control design avoided	3. Prevent inappropriate exclusions	4. Interpretation of index results without knowledge of the standard	5. Pre-specificied threshold used	6. Appropriateness of reference standard in classifying identified	7. Interpretation of reference results without knowledge of standard	8. Ideal interval between index and reference results?	9. Same reference results for all patients?	10. Inclusion of all paitents in analysis	Total score
Leighton et al., 2019	+	++	+	NA	++	++	NA	NA	++	+	4
Li et al., 2021	+	++	+	NA	++	++	NA	NA	++	++	5
Lin et al., 2021	+	++	+	NA	++	++	NA	NA	++	++	5
Lin et al., 2021	+	++	+	NA	++	++	NA	NA	++	++	5
Liu et al., 2022	+	++	++	NA	++	++	NA	NA	++	++	6
Magrangeas et al., 2022	++	++	+	NA	++	++	NA	NA	++	++	6
Modai et al., 1995	++	++	+	NA	++	++	NA	NA	++	++	6

Bias / Authors	1. Consecutive or random sampling	2. Case control design avoided	3. Prevent inappropriate exclusions	4. Interpretation of index results without knowledge of the standard	5. Pre-specificied threshold used	6. Appropriateness of reference standard in classifying identified	7. Interpretation of reference results without knowledge of standard	8. Ideal interval between index and reference results?	9. Same reference results for all patients?	10. Inclusion of all paitents in analysis	Total score
Mourao-Miranda et al., 2012	++	++	++	NA	++	++	NA	NA	++	+	6
Nijs et al., 2021	++	++	++	NA	++	++	NA	NA	++	++	7
Podichetty et al., 2021	++	++	++	NA	++	++	NA	NA	++	+	6
Sarpal et al., 2016	+	++	+	NA	++	++	NA	NA	++	++	5
Schie, 2022	+	++	+	NA	++	++	NA	NA	++	++	5
Soldatos et al., 2022	+	++	+	NA	++	++	NA	NA	++	+	4
Smucny et al., 2020	+	++	++	NA	++	++	NA	NA	++	++	6

Bias / Authors	1. Consecutive or random sampling	2. Case control design avoided	3. Prevent inappropriate exclusions	4. Interpretation of index results without knowledge of the standard	5. Pre-specificied threshold used	6. Appropriateness of reference standard in classifying identified	7. Interpretation of reference results without knowledge of standard	8. Ideal interval between index and reference results?	9. Same reference results for all patients?	10. Inclusion of all paitents in analysis	Total score
Talpalaru et al., 2019	+	++	+	NA	++	++	NA	NA	++	-	4
Van Hooijdonk et al., 2023	+	-	+	NA	++	++	NA	NA	++	++	4
Wang et al., 2022	+	++	+	NA	++	++	NA	NA	++	++	5
Wu et al., 2020	++	++	-	NA	++	+	NA	NA	++	++	5
NA=blue (not applicable); No = red (-); Unclear = yellow (+); Yes = green (++)											

Supplementary S3. Details of included studies (expanded version)

Authors/Year<br>Country/<br>Setting	Participants	Variables measured	Clinical outcomes	Main findings
Ambrosen et al., 2020<br><br>Denmark<br><br>Inpatient and outpatient clinics	Participants with first episode of psychosis, antipsychotic-naïve, n=138<br><br>Healthy controls from community of Copenhagen, n=151	Cognition measured using multiple scales[a]. MRI, Copenhagen Psychophysiology Test Battery, data on maternal and paternal age at birth, gestational age in weeks, birth length and weight, and Apgar scores.	Short term treatment response: PANSS(Fup) – PANSS(baseline)/ PANSS(baseline), between 6 weeks to 6 months<br><br>Long term treatment response: binary categorical variable, according to prescription, mean assessment time was 6-16.9 years.	Best algorithms:<br>Diagnostic classification: ensemble of trees with Bayesian optimization<br>Long-term treatment response (classification): logistic regression for high-dimensional data<br>Short-term treatment response: SVM with L1 regularization
Blessing et al., 2019<br><br>China<br><br>Shanghai Mental Health Centre	Participants with schizophrenia or schizophreniform disorder, n=67	MRI data; functional connectivity	BPRS ≥ 35% reduction= responders	Anteromedial hippocampal functional connectivity with right superior frontal gyrus, right posterior insular-opercular cortex, left precentral and postcentral gyrus predicted response to treatment.
Cao et al., 2018<br><br>China	Participants with first episode of psychosis, antipsychotic-naïve, n=38	MRI data; functional connections of superior temporal cortex, measured	Positive symptoms and hallucination subscale of PANSS, reduction of at least 30% = responders	The prediction accuracy between responders and non-responders is 82.5% using correlational functional connections and 57.4% using mutual information of the blood oxygen level dependent signals. Functional

Authors/Year<br>Country/<br>Setting	Participants	Variables measured	Clinical outcomes	Main findings
Community and Beijing Hui-Long-Guan Hospital	Healthy controls from community, n=29	with correlations and mutual information between blood-oxygen-level dependent signals of superior temporal cortex versus other cortical areas		connectivity between superior temporal cortex and other cortical areas was a predictor
Cui et al., 2021<br><br>China<br><br>Hospital	Participants with schizophrenia, dataset 1, n=85, Participants with schizophrenia spectrum disorders, dataset 2, n=63	MRI data; functional connectivity (functional features) and features of grey matter (structural features)	PANSS, 30% reduction= responders	Twelve features (3 cortical features and 9 functional connections) remained in the prediction model. Functional connection includes temporal pole, superior temporal gyrus, supramarginal gyrus, LTG, lateral occipital cortex, temporal occipital fusiform cortex, precentral gyrus, inferior frontal gyrus, caudate, putamen, intracalcarine cortex, pallidum, brain stem and parahippocampal gyrus. Cortical features include precuneus, right cuneus, and inferior parietal lobule.
Cui et al., 2021<br><br>China<br><br>Xijing hospital	Participants with schizophrenia, and healthy control:<br>Dataset 1: n=100, n=92.	MRI data; thalamic features	PANSS, reduction of at least 30% = responders	4 features from 4019 radiomics features were identified.

Authors/Year<br>Country/<br>Setting	Participants	Variables measured	Clinical outcomes	Main findings
	Dataset 2: n=91, n=107			
Ebdrup et al., 2019<br><br>Denmark<br><br>Psychiatric hospitals and outpatient mental health centers	Participants with schizophrenia or schizoaffective psychosis, n=46<br><br>Healthy control, n=58	Neurocognitive test battery of 4 tests yielding 25 cognitive variables, Copenhagen Psychophysiology Test Battery and MRI data.	Symptom remission after six weeks using PANSS Andreasen	No variable predicted symptom remission after six weeks.
Fond et al., 2019<br><br>France<br><br>10 schizophrenia expert centres	Stable patients (no hospitalisation or treatment changes in past 8 weeks) with schizophrenia or schizoaffective disorder, n=315	Demographic data, social history, psychiatric history including therapy and pharmacological treatment,<br><br>Scales including CDSS, GAF, PANSS, YMRS, Childhood Trauma Questionnaire, Birchwood Insight Scale, Buss & Perry scores for physical	Psychotic relapse within 2 years, defined as a psychotic episode lasting at least 7 days	Predictive factors for relapse at 2 years includes psychotic relapse past year, high numbers of hospitalisation, taking first generation antipsychotic, higher antipsychotic doses, metabolic syndrome, CDSS, GAF, Buss & Perry anger score, PANSS for the positive and depressed subscales.

Authors/Year<br>Country/<br>Setting	Participants	Variables measured	Clinical outcomes	Main findings
		aggressiveness, anger.		
Homan et al., 2019<br><br>United States<br><br>Northwell Health, Zucker Hillside Hospital	Participants with psychotic disorders or bipolar disorder with psychotic features, n=82<br><br>Healthy controls, n=58	MRI data; nodal degree of structural similarity network	BPRS<br>Individual response was measured as a continuous measure using mixed models	Nodes that were implicated were primarily located in the (1) prefrontal cortices, (2) posterior cingulate cortex, and (3) the precentral, superior temporal and middle cingulate cortex.
Kottaram et al., 2019<br><br>Australia<br><br>Orygen Youth Health clinical sites	Patients with schizophrenia, n=11<br><br>First episode psychosis, n=19	Scales including BPRS, general cognitive ability assessed by the Wechsler Abbreviated Scale of Intelligence<br>MRI data	Psychotic symptom at 1 year using BPRS	In baseline resting state connectivity within the default mode network, predictive factors for <u>worsening positive symptoms at 1 year</u> include hyper-dynamism, hypo-connectivity while predictive factors for <u>worsening negative symptoms at 1 year</u> include hypo-dynamism and hyper-connectivity. Networks tend to be at the left hemisphere for negative symptoms and comprised many regions for positive symptoms.
Koutsouleris et al., 2016<br><br>Israel and 14 European countries	Participants aged 18-40 years old with schizophrenia, schizoaffective or	Demographic data, employment, educational history, social history,	GAF ≥ 65 (good)<br><br>GAF < 60-65 (poor)	Predictive factors for <u>poor outcomes</u> at <u>4 and 52 weeks</u> included: unemployment, unmet needs in CAN including relationship, activities, and psychological distress. <u>At 4 weeks</u>, other predictors included poor baseline educational

Authors/Year<br>Country/<br>Setting	Participants	Variables measured	Clinical outcomes	Main findings
50 mental health centres	schizophreniform disorder in the past 2 years.<br><br>Training, n=334<br>Testing, n=108	antipsychotics utilised.<br>Scales including PANSS, CAN.		status, haloperidol treatment, unmet needs in CAN's items including money, information, accommodation, and sexual expression. At 52 weeks, other factors included recurrent relapses, male, suicidality, educational difficulties, lower baseline scores for PANSS items positive symptoms, conceptual disorganisation, and hyperactivity.<br><br>Predictive factors for good outcomes at 52 weeks include greater GAF scores, remission likelihoods, positive MANSA scores for job, leisure, friendship, and health
Lamichhane et al., 2023<br><br>USA<br><br>Zucker Hillside Hospital, crosscheck database	Outpatients with schizophrenia, n=63	Information from mobile, including<br>- light exposure<br>- mean of audio amplitude<br>- length of phone conversations<br>- distance travelled<br>- accelerometer<br>- screen time	Relapse episode that required hospitalization, greater clinical care needs, or defined as increased in total BPRS rating.	Changes in conversation, volume, and distance travelled, were most predictive of relapse.
Leighton et al., 2019<br><br>United Kingdom	Participants with first episode psychosis.	Baseline, 6th month, 12th month<br>Demographic: age, gender, ethnicity,	Employment/ education status at 12th month	One-year outcomes for (1) employment/ education status, (2) point and (3) period remission, respectively were:<br>Accuracy: 0.85, 0.61, 0.63

Authors/Year<br>Country/<br>Setting	Participants	Variables measured	Clinical outcomes	Main findings
NHS Greater Glasgow & Clyde	Training, n=83<br>Validation, n=79	citizenship, education level, household composition, baseline functioning, parental status, marital status, accommodation, alcohol and drug use<br><br>Beck's Depression Inventory II<br>PANSS<br>HADS	Point remission: PANSS ≤ 3 / 7 using Andreasen PANSS remission criteria at 12th month<br><br>Period remission: PANSS ≤ 3 / 7 at 6th and 12th month	Sensitivity: 0.82, 0.58, 0.61<br>Specificity: 0.88, 0.67, 0.65<br>PPV: 0.82, 0.79, 0.65<br>NPV: 0.88, 0.42, 0.61<br>Chronological predictive factors respectively, were<br>(1) baseline functioning, living with spouse and children, having an education, and negative predictors were: PANSS for suspiciousness, hostility, delusions, and alcohol use.<br>(2) living with spouse and children, depression, PANSS score for excitement and negative predictors are: PANSS social withdrawal and rented accommodation<br>(3) PANSS score for excitement, living with spouse and children, PANSS for depression, poor rapport, having a relationship, having an education, ethnicity: white, living with family. Negative predictors were rented accommodation, PANSS for social withdrawal, somatic concern, difficulty in abstract thinking and unusual thought content.
Leighton et al., 2019<br><br>Scotland<br>England	Participants with first episode psychosis from National EDEN studies (n=1027),	Specific items of PANSS and Premorbid Adjustment Scale, GAF, duration of	PANSS at 6th and 12th month<br><br>GAF ≤ 65<br><br>Vocational recovery	One-year outcomes for (1) symptom recovery, (2) social recovery (3) vocational recovery, (4) quality of life for (Scottish and OPUS datasets), respectively were:

Authors/Year<br>Country/<br>Setting	Participants	Variables measured	Clinical outcomes	Main findings
Denmark<br><br>Inpatient and outpatient services	Scottish validation dataset (n=162) and OPUS trial (n=578).	untreated psychosis, education level, drug use, time spent doing leisure activities, employment and income, self-harm, ethnicity, gender, CDS, forensic history, housing, family history of psychosis, anxiety and depression.	Quality of life	Accuracy: (0.70, 0.62), (0.46- OPUS), (0.84, 0.68), (0.70, 0.59).<br>Sensitivity: (0.62, 0.61), (0.78- OPUS), (0.90, 0.58), (0.96, 0.88).<br>Specificity: (0.77, 0.63), (0.40- OPUS), (0.81, 0.73), (0.50, 0.30).<br>PPV: (0.73, 0.48), (0.18-OPUS), (0.77, 0.49), (0.64, 0.56)<br>NPV: (0.67, 0.74), (0.91-OPUS), (0.91, 0.79), (0.90, 0.71).<br><br>Positive predictors were GAF scores, higher education, staying in own or parents' home, being employed, no self-harm, good social support and insight.<br>Negative predictors were hallucinations, unusual thought content, adolescent social withdrawal, substance use.
Li et al., 2021<br><br>China<br><br>13 hospitals	Participants with schizophrenia, aged ⩾ 18 and taking antipsychotics, n=550	Demographic characteristics, antipsychotic use, prolactin blood. Scales including PANSS, CDSS, CGI, Medication Satisfaction Questionnaire, Drug Attitude	Better social functioning measured by ≥ 10 points increase in the Personal and Social Performance scale	Positive predictors at 3 months' for (1) social functioning included female, younger age, unmarried, employed, first episode, outpatient treatment, shorter duration of relapse, lesser relapse, shorter duration of current episode, lower baseline social functioning score, fewer comorbidities, more severe scores measured by PANSS, CDSS and CGI.

Authors/Year<br>Country/<br>Setting	Participants	Variables measured	Clinical outcomes	Main findings
		Inventory, Subjective well-being under neuroleptics, Simpson Angus scale, Barnes Akathisia, Abnormal Involuntary Movement Scale.		
Lin et al., 2021<br><br>Taiwan<br><br>2 hospitals	Participants with schizophrenia, aged 18 – 65 years old, n=302	Scales including PANSS-Positive item, Scale for the Assessments of Negative Symptoms, 17-item Hamilton Depression Rating Scale	Cognitive functioning measures processing speed, attention, memory, learning, memory, reasoning, problem solving, social cognition by scales including Wechsler Adult Intelligence Scale.<br><br>Functional outcomes measured by Quality of life Scale and GAF	Quality of life was best predicted with the Scale for the Assessments of Negative Symptoms and 17-item Hamilton Depression Rating Scale.<br><br>GAF was best predicted with PANSS-Positive item and the Scale for the Assessments of Negative Symptoms.<br><br>Cognitive tests that best predicted quality of life were WAIS-III digit symbol-coding, category fluency, social cognition, verbal and non-verbal working memory.<br><br>Cognitive tests that best predicted GAF were category fluency, d-Prime of blurred version, WAIS-III digit symbol-coding, verbal working memory, problem solving and reasoning.

Authors/Year<br>Country/<br>Setting	Participants	Variables measured	Clinical outcomes	Main findings
Lin et al., 2021<br><br>Taiwan<br><br>2 hospitals	Participants with schizophrenia, aged 18 – 65 years old, n=302	11 genetic variants from blood including AKT1 rs1130233,<br>COMT rs4680,<br>DISC1 rs821616,<br>DRD3 rs6280,<br>G72 rs1421292,<br>G72 rs2391191,<br>5-HT2A rs6311,<br>MET rs2237717,<br>MET rs41735,<br>MET rs42336,<br>TPH2 rs4570625	Functional outcomes measured by Quality of life Scale and GAF	M5 prime algorithm identified G72 rs2391191 and MET rs2237717 that built the best model to predict quality of life.<br><br>M5 prime algorithm identified AKT1 rs1130233 that built the best model to predict GAF.
Liu et al., 2022<br><br>China<br><br>Xijing Hospital	Participants with schizophrenia, n=38<br><br>Control, n=38	Dynamic changes of degree centrality measures using MRI data processed by The Data Processing & Analysis for Brain imaging and computed using an equation	Treatment outcomes classified as non-responders or responders defined as reduction >50% of PANSS scale.	As compared with controls, there were significant differences in degree centrality amongst participants with schizophrenia. Reduce degree centrality were found in sub-cortical gray matter structures: bilateral cerebellum, putamen, hippocampus, thalamus, caudate, and increased degree centrality in precuneus, anterior cingulate cortex, middle temporal gyrus, medial frontal gyrus, and superior, bilateral inferior and medial, frontal gyrus.<br><br>After antipsychotic treatment, changes in degree centrality in participants with

Authors/Year Country/ Setting	Participants	Variables measured	Clinical outcomes	Main findings
				schizophrenia correlate with PANSS changes. Significant –ve correlation in left putamen and right putamen.
Magrangeas et al., 2022<br><br>London<br><br>SLaM NHS Foundation Trust	Participants with schizophrenia, schizotypal and delusional disorders, aged 16 to 95 years old, n=9323	Demographic data, age of psychosis onset, psychotic symptoms.<br><br>Scales including Index of Multiple Deprivation.	Negative outcomes within 2 years defined as<br>(1) ≥ 22 days of intensive treatment<br>(2) > 2 antipsychotics<br>(3) involuntary admission	Predictors of negative outcomes within 2 years included younger age, black ethnicity, poorer neighbourhoods and in those who reported paranoia, auditory hallucinations, persecutory delusions, somatic passivity and thought interference.
Modai et al., 1995<br><br>Israel<br><br>Gehah Psychiatric Hospital	Participants who were admitted consecutively from 1988 to 1990, n=289	Sociodemographic data, social history, psychiatric history and treatment.	GAF improved ≥ 40 points= positive outcome, improvements < 40 points= negative outcome	Predictors of positive outcomes at 8 weeks included higher socioeconomic class, positive symptoms, receiving psychotherapy, electroconvulsive therapy, Clozapine, or noradrenergic antidepressants. Other factors are older age onset, high premorbid level, axis II diagnosis and frequent hospitalisation.<br><br>Predictors of negative outcomes at 8 weeks included negative symptoms, duration of last hospitalisation stay, low potency antipsychotics, requiring community aid, resistant depression, or OCD.<br><br>Factors that were non-significant included gender, family status, education history,

Authors/Year<br>Country/<br>Setting	Participants	Variables measured	Clinical outcomes	Main findings
				diagnosis of depression, precipitating factors triggering relapse, serotonergic antidepressants, and mood stabilisers.
Mourao-Miranda et al., 2012<br><br>United Kingdom<br><br>Mental health services.	Participants 16 to 65 years old with psychotic illness, n=100<br><br>Control was recruited via advertisements, n=91	MRI data	Course of illness using WHO Life Chart, symptoms<br>>6 months (continuous),<br>1 episode >6 months and all other episodes < 6 months (episodic), no long periods of remission or psychotic symptoms (intermediate).	Continuous course could be differentiated from episodic course (accuracy=70%) and healthy individuals (accuracy=67%). Anatomical regions that discriminated continuous course vs (episodic and control) included parahippocampal gyri, basal ganglia, cingulate and thalami.<br><br>Episodic course could not be differentiated from control (accuracy=54%).
Nijs et al., 2021<br><br>Netherlands<br>Belgium<br><br>Mental health services	Participants 16 to 50 years old with psychotic disorder, n=523	Demographics, psychiatric history, extrapyramidal symptoms, genetic variables, substance use history, accommodation, neurocognitive and social cognitive task scores.<br><br>Scales including GAF, PANSS,	Symptomatic outcome using Andreasen PANSS remission criteria, PANSS ≤ 3 / 7.<br><br>Global functioning GAF ≥ 65 (good), GAF < 65 (poor).	<u>Predictors of symptomatic outcome at 3 years</u> included older age, self-harm, PANSS for depression, lack of spontaneity, hallucinatory behaviours, suspiciousness, poor judgement, and insight,<br><br><u>Predictors of symptomatic outcome at 6 years</u> included lack of activity, PANSS for flat affect, motor retardation, lack of spontaneity, abstract thinking difficulty, emotional withdrawal, hallucinatory behaviours, delusions, unusual thought content, poor judgement, and insight

Authors/Year Country/ Setting	Participants	Variables measured	Clinical outcomes	Main findings
		Camberwell Assessment scale of Need Short Appraisal Schedule, Community Assessment of Psychic Experiences, Premorbid Adjustment Scale items		
Podichetty et al., 2021<br><br>Data from CATIE trial	1600 participants with schizophrenia	Structured clinical interview, neurocognitive battery, vital signs CGI, CDSS.	PANSS, reduction of at least 20% = responders	Top 10 predictive factors were poor attention, depression, preoccupation, volition impairment, abstract thinking difficulty, sterotyped thinking, anxiety, abnormal thought content, excitement and observed depression. Random Forest performed best as compared with logistic regression, naïve Bayes, and support vector machine.
Sarpal et al., 2016<br><br>United States<br><br>Zucker Hillside Hospital	Training cohort (schizophrenia spectrum disorder), n=41<br><br>Testing cohort (schizophrenia spectrum	MRI data; generating whole brain functional connectivity maps from striatal seed regions	CGI score of 1 or 2 and BPRS-A (conceptual disorganisation, grandiosity, unusual thought content, hallucinatory behaviours) ≤ 3	91 connections were associated with response to treatment. At baseline, greater connectivity with striatal subdivision at posterior regions, and lower striatal connectivity at frontal regions were associated with better treatment response.

Authors/Year<br>Country/<br>Setting	Participants	Variables measured	Clinical outcomes	Main findings
	disorder or bipolar with psychotic features), n=40			
Schie, 2022<br><br>14 European countries, Israel and Australia<br><br>27 general hospitals and clinics	Participants 18 and older with first episode schizophrenia, schizophreniform disorder or schizoaffective disorder, taking antipsychotic for <15 days in past year.<br><br>4 weeks: n=309<br>10 weeks: n=57	Demographic characteristics, diagnosis, drug and alcohol, physical examination, medication dosage, psychiatric comorbidity, cytokines, MINI, PANNS, Personal and Social Performance Scale, CGI, SWN, depression measured by CDSS, 39 cytokines, vascular endothelial growth factor (VEGF), C reactive protein, serum amyloid A, soluble intercellular adhesion molecule-	Treated with amisulpride x 4 weeks<br>→ if PANSS ≤ 3 / 7, considered remitted at 4 weeks using Andreasen PANSS remission criteria.<br>→ If not remitted → randomly assigned to amisulpride or olanzapine and remitted if PANSS ≤ 3 / 7, at 10 weeks.	Non remitted patients were more likely to be older, males, living alone, unemployed, with higher weight and greater substance use versus patients who achieved remission.<br><br>Demographic factors, symptoms, cytokines, PANSS and PSP scores performed better in model while other variables such as substance use, physical findings, medication dosage, CDSS, CGI, SWN, and MINI ratings did not perform better than chance.<br>Amongst cytokines, Cytokine IL-18 was a predictor<br><br><u>4 weeks' outcome</u> for (1) Demographics, (2) diagnosis, (3) cytokines, (4) PANSS, (5) PSP, (6) all variables, respectively were:<br>Accuracy: 0.59, 0.58, 0.58, 0.60, 0.58, 0.64<br>Sensitivity: 0.70, 0.73, 0.74, 0.72, 0.67, 0.85<br>Specificity: 0.40, 0.37, 0.32, 0.38, 0.42, 0.31<br><br>Models for MINI, lifestyle, somatic, treatments, CGI, CDSS and SWN modalities were insignificant.

Authors/Year<br>Country/<br>Setting	Participants	Variables measured	Clinical outcomes	Main findings
		1 and soluble vascular adhesion molecule-1.		10 weeks' outcome for (1) cytokines, (2) CDSS, (3) SWN, (4) all variables, respectively were:<br>Accuracy: 0.66, 0.62, 0.60, 0.63<br>Sensitivity: 0.60, 0.67, 0.53, 0.54<br>Specificity: 0.61, 0.57, 0.62, 0.69.<br>Cytokine IL-18 most predictive.
Soldatos et al., 2022<br><br>Greece<br><br>5 psychiatric hospitals	Recruited from Athens First Episode Psychosis Research Study<br><br>Athens, n=179<br><br>Copenhagen, n=101	Demographic data, psychiatric history, duration of untreated psychosis, education level, employment status, cannabis use, numbers of co-habitants.<br><br>Scales including PANSS, CGI, Personal and Social Performance Scale	Remission measured by the Andreasen PANSS remission criteria.	Predictive factors of non-remission at 4-6 weeks include PSP and GAF scores, PANSS scores for delusions, social avoidance, passive/apathetic social withdrawal, blunted affect, emotional withdrawal, poor rapport, delusions, lack of spontaneity, flow of conversation, judgement, and insight.
Smucny et al., 2020<br><br>United States	Participants with onset of psychosis in the past 2 years.<br>Psychosis, n=82	Evaluation of fronto-parietal brain regions with cognitive control-associated	'Non-improver' versus 'Improver' which is defined as > 20% decrease in BPRS.	Deep learning model (Accuracy 65.9% - 78.4%) outperformed traditional machine learning models.<br>The activation of the dorsolateral prefrontal cortex was the most predictive.

Authors/Year<br>Country/<br>Setting	Participants	Variables measured	Clinical outcomes	Main findings
University of California, Davis, Psychosis clinic	Healthy control, n=138	activations, captured by MRI data.<br>Participants were presented with probes and cues and were instructed to press a specific button if it was preceded by certain cue letter 'A'.		
Talpalaru et al., 2019<br><br>Northwestern University Schizophrenia Data and Software Tool (NUSDAST) dataset	Patients with schizophrenia, n=104<br><br>Healthy controls, n=63	Demographics, average cortical thickness defined by Automated Anatomical Labelling atlas.	3 subgroups:<br><br>1) high symptom<br><br>2) mainly positive symptom<br><br>3) mild symptom	Paracingulate gyri and the left anterior cingulate differentiate normal control and the rest of the groups.<br>Right insula, middle temporal gyri and left temporal poles of the superior temporal affected in groups 1 and 2.<br>Left insula affected in groups 2 and 3.<br>The left Heschl's gyrus and right superior temporal gyrus differences between high symptom and control.<br>Medial orbital part of the superior frontal gyrus and orbital part of the right inferior frontal gyrus differentiates between group 2 and control.

Authors/Year<br>Country/<br>Setting	Participants	Variables measured	Clinical outcomes	Main findings
				Paracingulate gyri and the right anterior cingulate differentiates between mild symptom and control.
Van Hooijdonk et al., 2023<br><br>Netherlands<br><br>36 mental health institutes	Patients with psychotic disorders, n=1136. Patients with treatment resistant schizophrenia, n=200	Sociodemographic data, clinical data, neurocognitive data, functioning variables, PAS, CTQ, familial and environmental data.	Treatment resistant schizophrenia versus responsive.	Amongst those aged 16 to 19, poor premorbid functioning was most predictive of treatment resistant schizophrenia. Other factors were, not being married, young age at illness onset, childhood sexual trauma, lower education level, greater use of substance, and staying in non-urban environments.
Wang et al., 2022<br><br>China<br><br>Peking University Sixth Hospital and Beijing Huilongguan Hospital	Participants with schizophrenia, treated with single atypical antipsychotic, mostly taking Risperidone or Clozapine, n=97	MRI and genotype data<br>Gray matter volume, cortical morphologies, amplitude of low-Frequency Fluctuation, regional homogeneity, functional connectivity.	PANSS reduction above 50% (responders) or below 50% (non-responders)	Top 10 predictors associated were grey matter volume, cortical thickness, aberrant amplitude low-frequency fluctuation, cortical thickness and volume, surface area, curvature and sulcal depth.
Wu et al., 2020<br><br>Taiwan	Patients with schizophrenic disorder aged 16 to 74 years old,	Demographics, psychiatric and medical clinical and	Treatment success as measured by absence of hospitalisation and mortality in 12 months.	Aripiprazole and Amisulpride were most recommended during the testing.<br>Predictors included age, numbers of hospitalisations, visits to E room and

Authors/Year<br>Country/<br>Setting	Participants	Variables measured	Clinical outcomes	Main findings
Taiwan's National Health Insurance Research Database	prescribed at least one antipsychotic<br>Train, n=22 601<br>Test, n=9676	pharmacological histories.		outpatient clinics, previous prescription of benzodiazepines, mood stabilisers and antiepileptic medication.

BPRS=Brief Psychotic Rating Scale; CDS=Calgary Depression Scale; CAN=Camberwell Assessment of Needs; CDSS=Calgary Depression Scale for Schizophrenia; CGI=Clinical Global Impression; CTQ=Childhood Trauma Questionnaire; Fup=follow up; GAF=Global Assessment of Functioning; HADS=Hospital Anxiety and Depression Scale; MANSA=Manchester Short Assessment of Quality of Life Scale; MINI=Mini International Neuropsychiatric Interview; NPV=Negative Predictive Value; PAS=Premorbid Adjustment Scale; PSP=Personal Social Performance; PANSS=Positive and Negative Syndrome Scale; PPV=Positive Predictive Value; SWN= Subjective Well-Being Under Neuroleptic Treatment Scale; YMRS=Young Mania Rating Scale

[a]Danish version of the National Adult Reading Test, Wechsler Adult Intelligence, Brief Assessment of Cognition in Schizophrenia, Cambridge Neuropsychological Test Automated Battery, Buschke Selective Reminding Test, Symbol Digit Modalities Test, Trial Making tests A & B, Wisconsin Card Sorting Test, Speed and Capacity of Language Processing Test

Supplementary S4. Predictive accuracy interpretation

**Predictive accuracy**	**Scorings**	**Interpretation**
Area under curve	⩾0.9	Outstanding
	0.80 to <0.90	Excellent
	0.7 to <0.8	Acceptable
	0.50 to <0.70	Poor
Accuracy/ Balanced accuracy	>90%	Very good
	70% – 90%	Good
	60% – 70%	Acceptable
	< 60%	Poor
Root Mean Square Error	The closer to zero	Better accuracy
